# Role of Rhizospheric Microbiota as a Bioremediation Tool for the Protection of Soil-Plant Systems from Microcystins Phytotoxicity and Mitigating Toxin-Related Health Risk

**DOI:** 10.3390/microorganisms9081747

**Published:** 2021-08-16

**Authors:** El Mahdi Redouane, Richard Mugani, Majida Lahrouni, José Carlos Martins, Soukaina El Amrani Zerrifi, Khalid Oufdou, Alexandre Campos, Vitor Vasconcelos, Brahim Oudra

**Affiliations:** 1 Water, Biodiversity and Climate Change Laboratory, Phycology, Biotechnology and Environmental Toxicology Research Unit, Faculty of Sciences Semlalia, Cadi Ayyad University, Av. Prince My Abdellah, P.O. Box 2390, 40000 Marrakech, Morocco; redouane.elmahdii@gmail.com (E.M.R.); richardmugani@gmail.com (R.M.); majidalahrouni@gmail.com (M.L.); soukainaelamranizerrifi@gmail.com (S.E.A.Z.); oudra@uca.ac.ma (B.O.); 2 CIIMAR, Interdisciplinary Centre of Marine and Environmental Research, Terminal de Cruzeiros do Porto de Leixões, Av. General Norton de Matos, s/n, 4450-208 Porto, Portugal; jmartins@ciimar.up.pt (J.C.M.); acampos@ciimar.up.pt (A.C.); 3 Laboratory of Microbial Biotechnologies, Agrosciences and Environment, Department of Biology, Faculty of Science Semlalia, Cadi Ayyad University, P.O. Box 2390, 40000 Marrakech, Morocco; oufdou@uca.ac.ma; 4 Department of Biology, Faculty of Sciences, University of Porto, Rua do Campo Alegre, 4169-007 Porto, Portugal

**Keywords:** microcystins, *Vicia faba*, *Triticum aestivum*, rhizospheric microbiota, plant growth, microcystins bioaccumulation, risk assessment

## Abstract

Frequent toxic cyanoblooms in eutrophic freshwaters produce various cyanotoxins such as the monocyclic heptapeptides microcystins (MCs), known as deleterious compounds to plant growth and human health. Recently, MCs are a recurrent worldwide sanitary problem in irrigation waters and farmland soils due to their transfer and accumulation in the edible tissues of vegetable produce. In such cases, studies about the persistence and removal of MCs in soil are scarce and not fully investigated. In this study, we carried out a greenhouse trial on two crop species: faba bean (*Vicia faba* var. *Alfia 321*) and common wheat (*Triticum aestivum* var. *Achtar*) that were grown in sterile (microorganism-free soil) and non-sterile (microorganism-rich soil) soils and subjected to MC-induced stress at 100 µg equivalent MC-LR L^−1^. The experimentation aimed to assess the prominent role of native rhizospheric microbiota in mitigating the phytotoxic impact of MCs on plant growth and reducing their accumulation in both soils and plant tissues. Moreover, we attempted to evaluate the health risk related to the consumption of MC-polluted plants for humans and cattle by determining the estimated daily intake (EDI) and health risk quotient (RQ) of MCs in these plants. Biodegradation was liable to be the main removal pathway of the toxin in the soil; and therefore, bulk soil (unplanted soil), as well as rhizospheric soil (planted soil), were used in this experiment to evaluate the accumulation of MCs in the presence and absence of microorganisms (sterile and non-sterile soils). The data obtained in this study showed that MCs had no significant effects on growth indicators of faba bean and common wheat plants in non-sterile soil as compared to the control group. In contrast, plants grown in sterile soil showed a significant decrease in growth parameters as compared to the control. These results suggest that MCs were highly bioavailable to the plants, resulting in severe growth impairments in the absence of native rhizospheric microbiota. Likewise, MCs were more accumulated in sterile soil and more bioconcentrated in root and shoot tissues of plants grown within when compared to non-sterile soil. Thereby, the EDI of MCs in plants grown in sterile soil was more beyond the tolerable daily intake recommended for both humans and cattle. The risk level was more pronounced in plants from the sterile soil than those from the non-sterile one. These findings suggest that microbial activity, eventually MC-biodegradation, is a crucial bioremediation tool to remove and prevent MCs from entering the agricultural food chain.

## 1. Introduction

In the past few decades, cyanoblooms have been taking place with increasing intensity, prevalence, and toxicity in eutrophic freshwater ecosystems worldwide [1,2]. The profuse proliferation of bloom-forming cyanobacteria has been multi-decadal anthropogenic out-turn of both global warming and eutrophication [1,3]. Cyanoblooms thrive in eutrophic conditions triggered by high nutrient inputs from industrial/agricultural facilities and water-treatment plants. These facilities have been indiscriminately loading nutrient-rich wastes to surface waters [4,5]. Cyanoblooms have been long considered as a major threat to human, livestock, and plant health due to the toxic cyanopeptides, mainly microcystins (MCs), that they produce and release into the water bodies used for drinking, recreational, and irrigation purposes [6,7]. MCs are regarded as the most ubiquitous and commonly occurring group of cyanotoxins in freshwater systems [8], produced by several bloom-forming cyanobacteria belonging to the genera: *Microcystis*, *Planktothrix*, *Dolichospermum* (previously *Anabaena*), *Nostoc*, and *Oscillatoria* [9,10]. They are known as potent hepatotoxic and potential tumor-inducing cyanopeptides, acting as inhibitors of serine/threonine-protein phosphatases as well as disruptors of intracellular homeostasis [6,11,12]. Human exposure to MCs primarily occurs through ingestion of contaminated drinking water or aqua- and agri-foods (oral route), body-contact recreation in MC-containing water (dermal route), and inhalation of the aerosolized toxin [6].

MCs are chemically stable and recalcitrant to various physical and chemical degrading factors, including sunlight, high temperatures, and extreme pH owing to their ring structure [13,14,15]. They are monocyclic heptapeptides with the general structure: cyclo-(D-Ala^1^-X^2^-D-Me-Asp^3^-Z^4^-Adda^5^-D-Glu^6^-Mdha^7^), where X (2) and Z (4) represent two highly variable proteinogenic L-amino acids; in addition to five non-proteinogenic amino acids: D-alanine, D-erythro-*β*-methyl-D-aspartic acid (D-Me-Asp), D-glutamic acid (D-Glu), *N*-methyldehydroalanine (Mdha), and the unusual *β*-amino acid 3-amino-9-methoxy-2,6,8-trimethyl-10-phenyldeca-4,6-dienoic acid (Adda) [16,17]. The adda moiety is crucial for MC toxicity, and it is present in all MC congeners [18]. Furthermore, microcystin-LR represents the most toxic and prevalent congener of MCs with leucine (L) in position 2 and arginine (R) in position 4, followed by microcystin-RR (arginine, arginine) and microcystin-YR (tyrosine, arginine) [19].

Following cyanobacterium-cell death and lysis, MCs are released into the extracellular environment in which they withstand natural degradation for several days, with a half-life ranging from 0.4 to 251 days [20,21,22] and total concentrations ranging from less than 1 to 29,000 µg L^−1^ in surface waters [10]. Moreover, MCs have been detected in drinking and irrigation waters sourced from groundwater, with the total content varying from 0.06 to 1.8 µg L^−1^ [23,24,25]. Thereby, the World Health Organization (WHO) has recommended a guideline value of about 1 µg L^−1^ and 12 µg L^−1^ of total MCs in drinking water, for long- and short-term exposures, respectively [26]. MCs pose a serious health hazard to humans, animals, and plants, not only in freshwater ecosystems but also in agroecosystems. When they are introduced into cropland soils, via contaminated irrigation water, MCs persist within and get adsorbed onto clay minerals over a relatively long time, with a half-life ranging from 3 to 17.8 days [27,28,29] and total content varying from 0.05 to 187 µg MCs kg^−1^ DW [25,29,30,31]. Moreover, the occurrence of MC-producing cyanobacteria has been confirmed in agricultural soils so far, which exacerbates the problematic issue of these toxic cyanopeptides in the agroecosystems and the consumption of their related vegetable produce [25].

MCs can reduce crop growth and productivity resulted from impairing various physiological functions, substantially: (a) hormone metabolism and translocation; (b) photosynthesis rate; and (c) nitrogen uptake and metabolism [32,33,34]. In addition, MC-induced stress can affect plant cell functioning by generating high levels of reactive oxygen species (ROS) responsible for genetic- and metabolism-related impairments, such as (a) lipid peroxidation; (b) protein oxidation; and (c) DNA damage [35,36,37,38]. To date, the mechanisms underlying MCs phytotoxicity are not fully investigated as well as their persistence and bioavailability in cropland soil. Indeed, researchers have started to investigate the role of native soil microbiota in the removal of MCs to mitigate their load in the plant-soil system; and thus, attenuate their toxicity and accumulation in plant tissues, including the edible parts [39,40]. In this light, our previous works have highlighted the role of rhizobia in Leguminosae protection against chronic exposure to MCs [41,42,43,44]. We also found evidence that the native microbiota of faba bean rhizosphere was capable of mitigating MCs adverse effects on plant growth and physiology, besides reducing their uptake and accumulation in plant biomass [44].

In this work, we aim to emphasize the contribution of native rhizospheric microbiota in alleviating MC-induced stress on faba bean (*V. faba* L., var. *Alfia 321*) and common wheat (*T. aestivum* L., var. *Achtar*) cultures exposed to MCs introduced to the plant-soil system via irrigation water. Thereby, plants were grown in sterile and non-sterile soils, and growth indicators were measured, besides toxin accumulation in rhizospheric soil and related plant tissues. Health risk assessment over the consumption of contaminated crop products on both humans and livestock was appraised as well. Furthermore, MC accumulation was also investigated in sterile and non-sterile plant-free soils. Therefore, this work highlights the urgent need to, further and fully, undergo the underlying pathways of MC removal via the use of native rhizospheric microbiota. These microorganisms may constitute environmentally relevant and cost-effective tools to lower MC charge in soil and preserve the homeostasis of the soil-plant system.

## 2. Materials and Methods

### 2.1. Bloom Sampling and Microcystins Quantification

Cyanobacterial bloom material was collected in October 2010 from Lalla Takerkoust lake-reservoir located at 35 km south-west of Marrakesh, Morocco (31°36′ N, 8°2′ W, 664 m), with a 27 µm mesh phytoplankton net. The chromatographic analysis (high-performance liquid chromatography—photodiode detection array) revealed the presence of MC-LR as a predominant variant. In addition, total MCs were quantified in bloom crude extract using protein phosphatase 2A inhibition (PP2A) assay according to the method described in Bouaïcha et al. (2001) [45]. The PPA2 analysis of the bloom sample revealed a total concentration of about 11.5 mg MC-LR equivalent g^−1^ DW [42].

### 2.2. Microcystins Extraction and Purification

The cyanobacterial crude extract containing MCs was prepared as described by El Khalloufi et al. (2013) [42]. Briefly, freeze-dried material of *Microcystis aeruginosa*-bloom was suspended in ultrapure water (10 mg dry cells in 1 mL ultrapure water), subjected to ultrasounds (42 kHz) for 5 min, and then centrifuged at 10,000× *g* for 10 min. The pellet was re-extracted twice as before and all supernatants were pooled and filtered through a glass fiber filter (GF/C, 47 mm) prior to the pre-purification procedure. Cell-free *Microcystis* crude extract was subjected to solid-phase extraction to pre-purify MCs and get rid of a broad spectrum of compounds that may exert a similar phytotoxic effect to that of MCs. Briefly, MCs in the crude extract were pre-purified on octadecyl-silica cartridges (LiChrolut^®^ RP-18, 40–63 µm, 1000 mg 6 mL, Sigma-Aldrich, St. Louis, MO, USA) conditioned with 5 mL methanol and 5 mL ultrapure water. Afterwards, MC molecules adsorbed on the sorbent beds were eluted with 70% aqueous methanol (*v/v*) after rinsing the cartridge with 10 mL of 20% aqueous methanol (*v/v*). The aqueous methanolic eluate was vacuum-dried at 40 °C, resuspended in 1 mL of ultrapure water, and then stored at −20 °C [46]. The extract was used as a stock solution to prepare MC-containing irrigation water at the concentration of 100 µg MC-LR equivalent L^−1^ (an environmentally relevant concentration that was detected in Lalla Takerkoust reservoir, Marrakesh, Morocco). The artificially contaminated water was used to irrigate faba bean and common wheat plants during the greenhouse trial.

### 2.3. Experimental Setup

#### 2.3.1. Soil Collecting and Characterization

**Figure 1:**
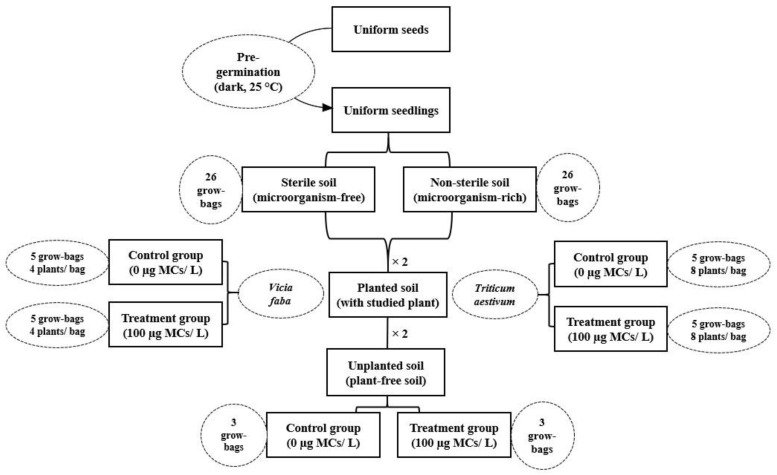
Experimental setup chart (×2): The design chart for planted and unplanted soils is the same either in sterile or non-sterile soils for each of the studied species.

15 subsamples of topsoil were randomly collected, in March 2019, at 20 cm depth from a cropland located in Lalla Takerkoust town (Marrakesh, Morocco) (31°22′ N, 8°7′ W, 612 m). The soil within was microcystin-free, as proven by an enzyme-linked immunosorbent assay, and had no history of any antecedent use of chemicals and pesticides that might impair or disrupt the development of native microbiota. Topsoil subsamples were air-dried at ambient temperature and passed through a 2 mm sieve to sift out pebbles and large debris, and then they were thoroughly mixed into one composite soil. Afterwards, the homogenized soil was divided into two subsets: one remained intact as microorganism-rich soil (non-sterile soil) and the other half was sterilized, at 150 °C in furnace muffle for 4 h, as microorganism-free soil (sterile soil). Sterile and non-sterile soils were transferred to 52 black plastic grow-bags (145 + 145 × 380 mm), each received 2.5 kg of soil: 26 bags for sterile soil and 26 bags for non-sterile soil (Figure 1).

The physicochemical and biological features of the soil are shown in Table 1. It was a silty clay loam soil comprising 7.03% sand, 64.5% silt, and 28.92% clay. The clay fraction consisted mainly of 50% hartite, 20% wollastonite, and 11% laumonite. It was slightly alkaline (pH—8.17) and non-saline soil (EC—0.42 dS. m^−1^); it contained 3.8 g organic matter kg^−1^ and 0.48 mg humic acids g-1. As for macronutrients, total nitrogen (N), phosphorus (P) and potassium (K) were 0.74, 0.42, and 24.57 g kg^−1^, respectively. Moreover, soil moisture (12.65%) and water-holding capacity (34%) were determined immediately following soil sampling. The total bacterial count was 3.28 × 10^7^ cfu g (dry weight) soil^−1^.

#### 2.3.2. Plant Culture and Exposure Experiment

The greenhouse trial was carried out in the Faculty of Sciences Semlalia, Cadi Ayyad University, Marrakesh, Morocco, from April to May 2019. Certified seeds of common wheat (*T. aestivum* L., var. *Achtar*) and faba bean (*V. faba* L., var. *Alfia 321*) were purchased from the national seed-marketing company (SONACOS-Société Nationale de Commercialisation de Semences, Marrakesh, Morocco) for the greenhouse trial. Uniform seeds were surface disinfected in 6% sodium hypochlorite solution for 10 min, washed thoroughly several times in sterile deionized water, and soaked in the last wash water for a few hours. Following this step, water-imbibed seeds were dark-germinated at 25 °C, in sterile glass Petri dishes (inner diameter, 150 mm) on a filter paper. Uniform seedlings were transferred into black plastic grow-bags containing either sterile or non-sterile soils (planted soils). Seedlings of each species were irrigated with MC-free water for one week after being germinated and then with MC-contaminated water (100 µg MC-LR equivalent L^−1^) for 30 days. Plants were irrigated, at 2-day intervals, with 100 mL of MC-free (control) and contaminated (treatment) waters, depending on their need for water and to maintain soil to its field capacity. Moreover, sterile and non-sterile bulk soils (unplanted soils) were also irrigated following the same aforementioned experimental setup similar to planted soil. The exposure experiments were arranged in randomized complete block design with each grow-bag (4 seedlings of faba bean/bag; 8 seedlings of common wheat/bag) and replicated 5 times for planted soil and 3 times for unplanted soil (Figure 1). The greenhouse experiment was carried out under environmental conditions of humidity, temperature, sunlight, and photoperiod of Marrakesh city.

### 2.4. Plant Harvest and Growth Indicators’ Determination

Before harvest, plants of each treatment were used for counting total leaf number (TLN) and measuring stem length (SL). At the final harvest, plants were uprooted and then roots were rinsed thoroughly under tap water to remove soil particles. Soon after harvest, we counted the total lateral root number (LRN) and fasciculated root number (FRN) of faba bean and common wheat plants, respectively. After that, root length (RL) was determined by measuring the length of the dominant root in faba bean plants and the mean length of five fasciculated roots of common wheat plants. Subsequently, plants were divided into two subsets of shoots and roots. One subset was oven-dried at 80 °C until constant weight, for measuring shoot and root dry weights (SDW and RDW respectively); the remaining subset was frozen in liquid nitrogen and freeze-dried for MCs quantification.

### 2.5. Determination of Microcystins in Plant Tissues and Soil

Enzyme-linked immunosorbent assay (ELISA) was used to determine total MCs in plant and soil matrices since chromatographic quantification is prone to under-report total MCs due to method specificity and presence of multiple and various congeners of MCs. Extraction and pre-purification of MCs from plants and soil were handled using dark glass vials and aluminum foil to avoid toxin photolysis. Only glassware was used during the extraction and the pre-purification procedures to avoid toxin adsorption on plastic material.

MC extraction from plant samples was conducted as described by Bavithra et al. (2020) [47]. Freeze-dried roots and shoots were ground into fine powder in liquid nitrogen. Following this step, aliquots of 250 mg of roots and shoots powder were homogenized with 25 mL of 50% aqueous methanol (*v/v*) (10 mg powder per 1 mL) and ultrasonicated (60 Hz) in an ice bath for 5 min using a probe sonicator (Hielscher, Teltow, Germany). The resulting slurry was kept at 4 °C overnight to increase the recovery of the toxin and then centrifuged (6000× *g*) at 4 °C for 10 min. The remaining pellets were re-extracted twice as before for a maximum yield of MCs. Afterwards, the cell-free supernatants of roots and shoots tissues were pooled together and MCs within were pre-purified on Supelclean octadecyl-silica cartridges (Supelclean^TM^ LC-18 SPE tube, 100 mg bed/1 mL, Sigma-Aldrich, Schnelldorf, Germany) conditioned with 5 mL methanol and 5 mL ultrapure water. MCs were eluted with 3 mL pure methanol after rinsing the cartridge with 10 mL of 20% aqueous methanol (*v/v*) and then the eluate was vacuum-dried at 40 °C. Afterwards, the residue was resuspended in 1 mL of ultrapure water and stored at −20 °C until ELISA analysis.

Composite samples of sterile and non-sterile bulk and rhizospheric soils were collected from each grow-bag using a sterile spatula, further freeze-dried, sieved at 2 mm, and ground with a mortar and pestle prior to MC extraction. MC extraction from soil samples was carried out as described by Chen et al. (2006) [48] and slightly modified. Briefly, aliquots of 2 g of lyophilized soil were extracted 3 times with 15 mL of a mixture solution containing 0.1 M ethylenediaminetetraacetic acid (EDTA), 0.1 M sodium pyrophosphate, and 0.002 M trifluoroacetic acid (TFA). The resulting slurry was subjected to ultrasonication (60 Hz) in an ice bath for 10 min and then left overnight at 4 °C. Following this step, the extract was centrifuged (6000× *g*) at 4 °C for 10 min. The pellets were re-extracted twice as before and then supernatants were gathered into one soil extract. MCs in the soil extract were pre-purified on Supelclean octadecyl-silica cartridges (Supelclean^TM^ LC-18 SPE tube, 100 mg bed/1 mL, Sigma-Aldrich, Schnelldorf, Germany) following the same procedure described above for plant extracts.

Total MCs were quantified by ELISA using the Eurofins Abraxis Microcystins-ADDA ELISA kit (Warminster, PA, USA) specific for the detection of microcystins and nodularins, with a limit of detection = 0.1 µg MC equivalent L^−1^. The ELISA analysis was run following the procedure described in the user’s guide provided along with the kit. The optical density was read at 450 nm using a Multiskan FC microplate photometer from Thermo Fischer Scientific (Strasbourg, France). A certified standard of MC-LR was used to construct the calibration curve at concentrations of 0.15, 0.4, 1, 2, and 5 µg L^−1^. Samples and standards were analyzed in triplicate and the results regarding MC concentrations were expressed as micrograms of MCs equivalent per kilogram of the dry weight of tissue or soil (µg kg^−1^ DW).

### 2.6. Evaluation of Health Risk

#### 2.6.1. Bioaccumulation Factor and Bioconcentration Level

The bioaccumulation factor (BAF) was used to evaluate MC accumulation in plant tissues using the Equation (1) given below [49]:

BAF = C_plant_/C_water_ (1)

where C_plant_ and C_water_ are MC concentrations in plant tissues (shoot and roots; µg kg^−1^ DW) and irrigation water (µg L^−1^), respectively.

MC bioconcentration level (BL) was appraised based on BAF values; 0 ≤ BAF < 3 indicates a low MCs bioconcentration in plant tissues; 3 ≤ BAF ≤ 3.7 represents a moderate MC bioconcentration; and BAF > 3.7 indicates a high MC bioconcentration.

#### 2.6.2. Estimated Daily Intake and Health Risk Quotient

To appraise the health risk related to irrigating faba bean and common wheat with MC-containing water, the estimated daily intake (EDI) of MCs in each plant was determined (µg MCs per kg of body weight per day) for both humans and farm animals, by the Equation (2) given below [50]:

EDI = (C_shoot_ × D_food intake_)/B_average weight_ (2)

where C_shoot_, D_food intake_, and B_average weight_ represent MCs concentration in shoots (µg kg^−1^ DW), average daily intake of tissue consumed (kg), and average body weight (kg), respectively. An average-sized adult with 60 kg body weight, in Morocco, was considered to consume 0.34 g and 500 g of beans and wheat (mean values), respectively, on a daily basis according to the data obtained from the Food and Agriculture Organization of the United Nations [51]. The content of MCs in the shoot part was used to determine the EDI of MCs in seeds of both species as a matter of supposition. The EDI results were compared to the tolerable daily intake threshold (TDI) set by the World Health Organization (WHO) to 0.04 µg MC-LR equivalent per kg body weight per day (µg kg^−1^ bw d^−1^) [52].

Shoots of leguminous and cereal crops are widely used as silage (faba bean) and straw (common wheat) to feed farm animals such as cattle. MC intake by livestock is not widely investigated to establish a threshold concentration whose exceeding may pose a sanitary hazard to farm animals when consuming MC-contaminated fodder on a daily basis. Nevertheless, increasing sanitary risk to livestock is prone to occur when exceeding 4.2 µg MCs equivalent L^−1^ in drinking water, for cattle weighing 800 kg on average, and drinking 85 L of water daily, according to the Australian and New Zealand Environmental and Conservation Council (ANZECC) [53]. Thereby, the TDI of MCs for cattle is around 0.45 µg per kg of body weight per day. The EDI for cattle was calculated using Equation (1), in which an average-sized animal of 800 kg of body weight consumes 32 kg of faba bean silage or wheat straw on a daily basis [44].

The health risk quotient (RQ) regarding the consumption of MC-polluted crops was evaluated according to Equation (3) [40] and it represents the factor exceeding the tolerable daily intake (TDI):

RQ = EDI/TDI(3)

where TDI indicates daily reference dose for humans (0.04 µg kg person^−1^ d^−1^) and cattle (0.45 µg kg^−1^ bw d^−1^).

A risk level was assessed based on RQ values; RQ > 1 indicates a high health risk, 0.1 ≤ RQ ≤ 1 represents a moderate health risk, and RQ < 0.1 indicates a low health risk [40].

### 2.7. Statistical Analysis

The experimental design was a randomized complete block. Data regarding the growth indicators related to TLN, SL, SDW, LRN, FRN, RL, and RDW were means of 10 replicates per treatment and parameters related to the MC accumulation in soil and plant tissues (shoots and roots), and health risk assessment (EDI, BAF, and RQ) were means of three replicates per treatment. Data were analyzed by variance analysis (ANOVA) and the mean separation was achieved by LSD test using the COSTAT software. Differences were considered significant at the probability level of *p* < 0.05.

## 3. Results

### 3.1. Effects of Microcystins on Plant Growth and Morphology

During the 30-day exposure to MCs at 100 µg L^−1^, faba bean and common wheat did not show visible morphological changes (leaf chlorosis and necrosis) in both sterile and non-sterile soils, in comparison to the control group. Root and shoot growth indicators (TLN, LRN, FRN, SL, RL, SDW, and RDW) were measured and shown in Table 2. Overall, MCs significantly reduced (*p* < 0.05) the growth indicators of faba bean and common wheat when grown in sterile soil compared to non-sterile soil where no significant decrease was observed between the control and the treatment groups. TLN exhibited no significant change in both faba bean and common wheat plants grown in sterile and non-sterile soils. However, SL, SDW, LRN, RL, and RDW of faba bean grown in sterile soil had significantly decreased by 16.9%, 22.39%, 34.71%, 24.45%, and 33.1% compared to the control, respectively. Likewise, SL, SDW, and RL of common wheat, cultivated in sterile soil, were remarkably reduced by 13%, 33.05%, and 15.6%, respectively; whereas FRN and RDW showed no significant changes compared to the control group.

MCs, in sterile soil, appeared to be more detrimental to faba bean roots than shoots, where RL and RDW decline were more pronounced than SL and SDW in the studied species. As for non-sterile soil, plants grown jointly with native rhizospheric microbiota and irrigated with MCs extract showed a similar growth rate to the control group; the observed growth decreases were not significant at 0.05 level. These findings suggested that plants were more protected from the phytotoxic effects of MCs in soil-based culture. That is to say, the soil was populated by microorganisms that may contribute to MC removal through a microbially mediated degradation process. Therefore, faba bean and common wheat plants displayed fewer growth impairments in the presence of rhizospheric microbiota compared to those grown in microorganism-free soil.

### 3.2. Accumulation of Microcystins in Soil-Plant System

MCs were accumulated differently among studied species (*V. faba* and *T. aestivum*) and plant organs (stem/leaves and roots) in sterile and non-sterile soils, and within rhizospheric and bulk soils (sterile and non-sterile) upon 30-days exposure to these cyanotoxins. The results obtained here and depicted in Table 3 demonstrated that MCs were less accumulated in non-sterile soil (bulk and rhizospheric soils) and in plant tissues of faba bean and common wheat grown within. Although MC content in faba bean shoots was almost the same in both plants grown in sterile and non-sterile rhizospheric soils.

We noticed that the highest concentrations of MCs were detected in shoot tissues of common wheat with total MC content of about 88.12 and 60 µg kg^−1^; and faba bean roots with total MC content of up to 49.13 and 20.38 µg kg^−1^, in sterile and non-sterile soils, respectively. The MC content increased sixfold and threefold in roots compared to shoots of faba bean plants grown in sterile and non-sterile soils, respectively; while it increased sevenfold and twelvefold in shoots compared to roots of common wheat plants cultivated in sterile and non-sterile soils, respectively. As for the soil compartment, the highest concentrations of MCs were found in sterile rhizospheric soil of faba bean (0.187 µg kg^−1^) and sterile bulk soil (0.161 µg kg^−1^); while the lowest concentration was detected in non-sterile rhizospheric soil of common wheat (0.08 µg kg^−1^).

Moreover, the bioaccumulation factor (BAF) of MCs was calculated to evaluate their bioconcentration level in plant tissues after a 30-day exposure experiment to 100 µg L^−1^ of MCs. As shown in Table 3, all BAF values were between 0 and 3, indicating a low bioconcentration level of MCs in shoots and roots of the studied species. Overall, MCs were more accumulated in the soil-plant system devoid of microorganisms (sterile soil) compared to the system replete with microorganisms (non-sterile soil). These findings suggest that in the presence of soil native microbiota, in bulk and rhizospheric soils, a part of the MCs might be removed by their degraders, which could result in less accumulation of the toxin in plant tissues.

### 3.3. Health Risk Assessment

We investigated and assessed the health risk over the consumption of MC-containing plants upon chronic exposure to MCs at 100 µg L^−1^. We calculated the EDI, according to the average amount of the plant consumed by humans and cattle (see Section 2.6.2), and the RQ to evaluate the risk level related to these cyanotoxins. We found evidence that plants grown in sterile and non-sterile soils posed a sanitary biohazard to both humans and cattle over the consumption of the contaminated tissues as shown in Table 4. For humans, the EDI of MCs exceeded the TDI set by the WHO at 0.04 µg kg^−1^ day^−1^, by an 18- and 12-fold increase in common wheat plants grown in sterile and non-sterile soils, respectively. These results indicated a high-risk level revealed by RQ values that were far beyond 1 for all plants grown in sterile and non-sterile soils. Although, the EDI values were bigger for common wheat plants cultured in sterile soil (EDI = 0.73 µg kg person^−1^ day^−1^) compared to those of the non-sterile soil (EDI = 0.5 µg kg person^−1^ day^−1^). As for faba bean plants, the EDI did not reach or transcend the TDI set for humans in both plants of the sterile (EDI = 4.3 ×10^−3^ µg kg person^−1^ day^−1^) and non-sterile (4 ×10^−3^ µg kg person^−1^ day^−1^) soils. However, these results indicated a moderate-risk level, related to the consumption of the leguminous plant, with RQ values between 0.1 and 1.

As for cattle, all EDI values exceeded the TDI set at 0.045 µg kg^−1^ (live weight) day^−1^ by ANZECC. The EDI was reduced in faba bean plants from the non-sterile soil compared to the sterile one with values of about 0.28 and 0.31 µg kg^−1^ (live weight) day^−1^, respectively. These values exceeded the TDI set for cattle and indicated a moderate-risk level evaluated through RQ values. Likewise, the EDI was decreased in common wheat plants grown in non-sterile soil compared to the sterile one with values of about 2.4 and 3.52 µg kg^−1^ (live weight) day^−1^, respectively. These results revealed a high-risk level over the consumption of common wheat; where RQ values indicated a sevenfold and fivefold increase of MCs in shoot tissues of plants from sterile and non-sterile soils, respectively. Overall, common wheat represented the plant with the highest sanitary risk for both human and cattle. Moreover, the EDI and the RQ values were decreased in non-sterile soil; hence the role of rhizospheric bacteria in minimizing MCs biohazard in edible crops.

## 4. Discussion

### 4.1. Impact of Microcystins on Plants’ Morphology

Exposure of faba bean and common wheat crops to MCs at 100 µg L^−1^ showed no observable toxicity symptoms like chlorosis or necrosis. Likewise, in our previous studies, growth and leaf development of faba bean (*V. faba*) variants (Aguadulce and Alfia 5) were similar to the control group upon 30-day exposure to total MCs at 2500 and 100 µg L^−1^ respectively; no leaf chlorosis or necrosis were perceptible [44,54]. However, MC-induced chlorosis and leaf size alterations have been previously reported by Pflugmacher et al. (2007) [55], where spinach (*Spinacia oleracea*) cultivars showed more chloroses and smaller leaves compared with control conditions, upon exposure to MCs at 0.5 µg L^−1^ over 42 days. Moreover, in a previous study carried out by Llana-Ruiz-Cabello et al. (2019) [56], pure MC-LR caused severe leaf necrosis in spinach plants at 50 µg L^−1^. On the same note, Zhu et al. (2018) [57] have perceived leaf chlorosis and necrosis in cucumber (*Cucumis sativus*) plants as well as root- and shoot-size decreases at 100 and 1000 µg L^−1^ MCs after only 7-days exposure.

### 4.2. Impact of Microcystins on Plants’ Growth in Absence of Native Rhizospheric Microbiota

The root system of the studied species displayed a high sensitivity to MCs in sterile soil compared to the control group. LRN and FRN of faba bean and common wheat plants were significantly decreased by 34.71% and 14.29%, respectively, under MC exposure. In a previous study conducted by Saqrane et al. (2008) [58], the lateral root number of lens (*Lens esculenta*), corn (*Zea mays*), durum wheat (*T. durum*), and pea (*Pisum sativum*) were reduced, under MC exposure at 11.6 µg mL^−1^, by 43%, 55%, 67%, and 99%, respectively. Additionally, Cao et al. (2018) [59] reported that crown root number and lateral root number of rice plants decreased by 33% and 37%, respectively, at 500 µg L^−1^ of MCs. It was also evidenced that MCs, at 4000 µg L^−1^, reduced the number of lateral root primordia and crown roots in rice by 72% and 47% compared to the control, respectively [60].

Moreover, RL and RDW were reduced up to 24.45% and 33.1% in faba bean culture and up to 14.29% and 26.98% in common wheat culture, respectively. These results were in the same line with our previously reported data in which RL and RDW of faba bean (Aguadulce cultivar), grown in inoculated (one rhizobial strain) sterile sand, exhibited a significant decrease up to 19% and 53%, respectively, upon exposure to 100 µg L^−1^ MCs during 35 days [42]. However, we have previously reported that faba bean (Alfia 5 cultivar) exposure to MCs at 2.5 mg L^−1^ did not result in a significant decrease in RDW in sterile soil [44]. Yet, cultivars of the same species (e.g., radish and lettuce) showed different biomasses in response to soil contaminants like heavy metals and organic pollutants [61,62]. We have also recorded a significant decrease in the RDW of alfalfa (*Medicago sativa*) seedlings cultivated in inoculated (one rhizobial strain) sterile sand under MC exposure in the range of 5 to 20 µg L^−1^ of MCs [42]. In this study, roots were significantly impaired compared to the shoot organs since they constitute the first organs that come in direct contact with MCs in soil. In this light, MCs were found to impede the development of the primary root tissue of several crop plants such as pea (*P. sativum*) [58], faba bean (*V. faba*) [63], and common bean (*Phaseolus vulgaris*) [64], besides causing cytoskeletal alterations as it was observed in rice (*Oryza sativa*) root cells [65].

In this study, MCs did not impact the TLN of faba bean and common wheat compared to the control plants. A similar result has been reported in Lahrouni et al. (2013) [54], who evidenced no significant change in the TLN between treated (100 µg L^−1^ MCs) and untreated faba bean plants (Aguadulce cultivar) grown in inoculated sterile sand. In contrast to this result, Saqrane et al. (2009) [66] recorded a significant decrease in the TLN of leguminous species: pea (*P. sativum*) and lens (*L. esculenta*); and cereal species: durum wheat (*T. durum*) and corn (*Z. mays*) grown in hydroponics (sand-based culture). Moreover, the shoot growth was affected by MCs in sterile soil, where SL and SDW were decreased by 16.9% and 22.39% in faba bean plants and by 13% and 33.05% in common wheat plants, compared to the control, respectively. These results matched with our previous findings in which SL of faba bean (Alfia 5 cultivar) was reduced up to 30.96% in sterile soil; whereas SDW was only reduced by 7.75% (not significant at 0.05 level) [44]. Similarly, we have previously recorded, in two separate studies, a SDW decrease of about 27% [43] and 31% [54] in faba bean plants (Aguadulce cultivar) grown in inoculated sterile sand and exposed to 100 µg L^−1^ of total MCs. Furthermore, MCs reduced the SL and SDW in a range of leguminous plants such as pea (*P. sativum*) and lens (*L. esculenta*); and cereal plants such as durum wheat (*T. durum*) and corn (*Z. mays*), which were grown in the sand (microorganisms-poor substrate) [66]. Likewise, alfalfa (*M. sativa*) seedlings grown in inoculated sterile sand and irrigated with MCs, at the range of 5–20 µg L^−1^, showed less biomass compared to the control plants [42].

### 4.3. Impact of Microcystins on Plants’ Growth in Presence of Native Rhizospheric Microbiota

MCs at 100 µg L^−1^ were not detrimental to the growth process of both faba bean and common wheat in non-sterile soil in terms of all investigated growth indicators. Thus, native microorganisms of the cropland soil were apt to protect the two studied plants from the deleterious effects of MCs compared to those grown in sterile soil. These results were very much in line with our previous study, in which faba bean plants (Alfia 5 cultivar) cultivated in non-sterile soil were not different from the control group in terms of SL, SDW, and RDW, upon chronic exposure to MCs at a high dose up to 2.5 mg L^−1^ [44]. Furthermore, we have previously reported that MCs at 100 µg L^−1^ reduced RDW of faba bean (Aguadulce cultivar) by 37% in sterile sand-vermiculite; whereas the use of a symbiotically MC-tolerant *rhizobium* protected plants from MCs biohazard [43]. Indeed, the use of MC-tolerant rhizobial symbiosis has been proven efficient to boost the growth and physiological response of leguminous plants exposed to MCs stress [41,42,43]. However, MCs reduced plant growth of several non-leguminous species grown in non-sterile soil and exposed to a similar dose of MCs around 100 µg L^−1^ or even less, such as common wheat (*T. aestivum)*, carrot (*Daucus carota*), lettuce (*Lactuca sativa*), and radish (*Raphanus sativus*) [30,55,67].

These findings suggested that plants’ response to MCs was species- and cultivar-dependent; and it was, possibly, related to the presence or absence of MCs-degrading microorganisms in the rhizosphere compartment. In this study, we used an agricultural soil known for its long history of being irrigated with MC-containing water, from Lalla Takerkoust lake-reservoir (the soil was left unplanted over one year and was thus free of MCs at the time we used it). Thereby, it was likely that native microbiota, in the soil, evolved to protect crop plants from the harmful effects of MCs introduced via irrigation water. The acquisition of such a catabolic trait might occur through a horizontal gene transfer mechanism between native microbiota and MC degraders from MC-polluted waters. Moreover, it is quite possible that rhizospheric microorganisms with MC-degrading trait were sourced from MC-contaminated waterbodies and were, thus, introduced to the soil system via irrigation water. The results depicted in this study highlighted the protective role of rhizosphere microbiota against the adverse impact of cyanotoxins introduced into the soil-plant system via contaminated irrigation water. In absence of microorganisms, MCs were highly accumulated in soil and highly available to plants thereafter. Thereby, MCs may disrupt and impair various physiological functions entailed in biomass build-up such as photosynthesis [33,44] and regulation of plant growth and development such as phytohormones biosynthesis and translocation [34,68]. As this happened, plants showed a decrease in growth rate in terms of several growth indicators like those investigated in this study.

### 4.4. Bioaccumulation of Microcystins in Plants and Health Risk Assessment

There has been growing attention to MC bioaccumulation in edible crops in the past few years, as elucidated by several studies. Some of these studies have been conducted on cereal species such as corn (*Z. mays*), durum wheat (*T. durum*) [66] and rice (*O. sativa*) [29,69,70], and leguminous crops such as pea (*P. sativum*), lens (*L. esculenta*), faba bean (*V. faba* var. *Alfia 5*) [44,66], and green beans (*P. vulgaris*). We found evidence that MC concentrations were higher in roots of faba bean than in shoots, which was consistent with several previous studies conducted on leguminous species [30,44,66] and non-leguminous species [40,69,71,72]. The mechanisms that underlie MC uptake by the root system are not fully understood and investigated to date. However, it was suggested that MCs, as cyclic heptapeptides, are taken up by peptide transporters of the root cells and accumulated mostly in root tissues due to their limited translocation within [29,44].

The highest concentration of MCs detected in faba bean (Alfia 321) was 49.13 µg kg^−1^ in the root part of plants grown in sterile soil; while it was lower in non-sterile soil with the total MC concentration of about 20.38 µg kg^−1^. However, shoots of common wheat plants exhibited the highest concentration of MCs, up to 88.12 µg kg^−1^ in sterile soil and 60 µg kg^−1^ in non-sterile soil. These results were in line with our previous pot experiment carried on faba bean plants (Alfia 5) upon exposure to MCs at 2500 µg L^−1^ over 28 days; where shoots accumulated the highest concentration of MCs in sterile soil (13.1 µg kg^−1^) than in non-sterile soil (8 µg kg^−1^) [44]. Interestingly, we evidenced that MCs were more accumulated in shoots of common wheat than in roots; which was in disagreement with previous studies conducted on cereal species such as rice (*O. sativa*) and corn (*Z. mays*) [29,66] and non-cereal species such as green beans (*P. vulgaris*), carrot (*D. carota*), and lettuce (*L. sativa*) [30]. Nevertheless, Saqrane et al. (2009) [66] found out that the total MC content, in both stem and leaves of durum wheat (*T. durum*) and, pea (*P. sativum*), were higher than the root part upon 30-days exposure to MCs at 4200 µg L^−1^. It seems that MC bioaccumulation varied among crop species and cultivars, and between plant parts (shoots versus roots) and soil status (sterile versus non-sterile). Moreover, the bioaccumulation process may depend mostly on translocation mechanisms of MCs from an organ to another and within the different tissues as well.

The bioaccumulation factor (BAF) reflects the transfer of MCs from the extracellular environment (soil and water) to the intracellular compartment of the root cells and their translocation to the aerial organs thereafter [70]. The BAF values of faba bean and common wheat indicated a low bioconcentration level of MCs (0 ≤ BAF < 3) at 100 µg L^−1^ used in irrigation water. Likewise, in a previous study carried out by Zhu et al. (2018) [57], the bioconcentration level of MCs was low in cucumber fruits (*C. sativus*) irrigated with MC-containing water at 10, 100, and 1000 µg L^−1^ during the seedling, flowering, and fruiting stages. Although, several studies have recorded a high bioconcentration level of MCs in edible parts of various vegetable produce, including lettuce (*L. sativa*), celery (*Apium graveolens*), cabbage (*Brassica oleracea*), garlic (*Allium sativum*), and faba bean (*V. faba*) in which BAF values were in the range of 3.8–23.4 [31,44,73]. In this study, the BAF values of plants grown in non-sterile soil were lower than those of plants cultivated in sterile soil. These results were consistent with our previous study on faba bean (Alfia 5), where BAF of MCs were lower in plants grown in non-sterile soil compared to sterile soil [44].

Furthermore, we assessed the health risk related to the consumption of the studied species by measuring the EDI and RQ of the toxin for both humans and cattle. In our study, faba bean posed no sanitary risk to humans for both plants from the sterile and non-sterile cultures since the EDI did not exceed the tolerable daily intake (TDI) set by the WHO at 0.04 µg kg person^−1^ day^−1^. However, the EDI of MCs in common wheat exceeded the TDI by about 18-fold and 12-fold in plants grown in sterile and non-sterile soils, respectively. Based on this result, common wheat may pose a serious health hazard to humans since wheat-based food is widely consumed worldwide on a daily basis up to 183.67 g person^−1^ on average (world average) [51]. In a previous study carried out by Liang et al. (2020) [70], the EDI of MCs in rice grains exceeded the TDI (1.148 times) upon irrigation with the same dose of MCs as this study (100 µg L^−1^). Moreover, several studies have recorded values of EDI that exceeded the TDI 1.65–79.75 times such as lettuce (*L. sativa*), garlic (*A. sativum*), horse bean (*V. faba*), spinach (*Spinacia oleracea*), cabbage (*B. oleracea*), smooth amaranth (*Amaranthus hybridus*), radish (*R. sativus*) and carrot (*D. carota*) [29,50,72,74]. It is to emphasize that EDI values of MCs in sterile soil were higher than those of the non-sterile soil for both species for both humans and cattle, which was a consistent result with the findings previously reported for faba bean (Alfia 5) grown in sterile and non-sterile soils as well [44]. These findings emphasize the protective role of native microbiota in some agricultural soils in mitigating the sanitary risk of MCs when consuming MC-contaminated produce. However, it is to mention that only the extractable fraction of MCs (free MCs) was analyzed throughout these works; while the non-extractable fraction of the toxin is covalently bound in tissues and thus not considered when assessing the health risk related to MC-contaminated produce. In a study carried out by Corbel et al. (2016) [71], plants of tomato (*Solanum lycopersicum*) were exposed to radiolabeled ^14^C-MC-LR at 100 µg L^−1^ in a hydroponic system and the radioactive fraction of non-extractable MCs was higher than that of the extractable ones in roots and leaves reaching 56% and 71% of the total accumulated toxin. Therefore, covalently bound MCs should be monitored as well in further studies on vegetable produce using the 2-methyl-3-methoxy-4-phenylbutyric acid (MMPB) method to determine total MCs (free and bound) in plant tissues similarly to animal tissues [75].

Most of the previous studies have only considered appraising the health risk related to MCs ingestion for humans (adult and children), but not for farm animals such as dairy and beef cattle. Therefore, we attempted to assess the health risk over the consumption of MC-polluted fodder (silage, straw) for cattle. The data obtained throughout this work revealed EDI values over the recommended threshold limit set, for farm animals, at 0.045 µg kg^−1^ (live weight) d^−1^ [53]. Both species, faba bean, and common wheat, from sterile and non-sterile soils, showed a moderate- and a high-risk level related to their consumption, as the EDI values exceeded the TDI of MCs for cattle (0.68–7.83 times). The sanitary risk was more pronounced in plants grown in sterile soil than in non-sterile soil, which was in agreement with the results obtained in a previous study on faba bean plants (Alfia 5) grown in sterile and non-sterile soils as well [44]. In another study, Crush et al. (2008) [76] reported that clover plants, grazed by cattle, accumulated MCs up to 21 mg MCs kg^−1^ DW, which was beyond the TDI several times. Thereby, a global public health concern is growing regarding the potential entrance of MCs to the chain food via contaminated dairy and beef products. Yet, studies about the transfer and accumulation of MCs in animal products (e.g., meat and milk) are scarce and not well documented. Orr et al. (2001 and 2003) [77,78] have previously carried out a study on dairy and beef cattle, by supplying their drinking water with the cells (10^5^ cells L^−1^) of a MC-producing cyanobacterium (*Microcystis aeruginosa*). Based on the data obtained, no problematic amounts of MCs were detected in the analyzed milk (<2 ng L^−1^) and liver tissues that may present an unacceptable risk to human health. However, more studies have to be further conducted to fill the gap in the literature about the transfer and accumulation of MCs in products derived from farm animals. Moreover, threshold limit values have to be established for dairy and beef products that come from animals fed with MC-containing fodder in a similar way to those set for water and vegetable produce.

### 4.5. Accumulation and Potential Removal of Microcystins in Agricultural Soil

In the present study, we assessed the accumulation of MCs, not only in plant tissues but also in bulk (plant-free soil) and rhizospheric soils (planted soil), sterile and non-sterile. MCs released into the surface waters, during cyanobloom expansion and decay, are introduced to cropland soils in which they persist and get transferred to cultivated plants. In this study, MCs persisted in the soil after harvest and were at the range of 0.08–0.18 µg kg^−1^ DW and 0.11–0.16 µg kg^−1^ DW in rhizospheric and bulk soils, respectively. Overall, MCs detected in agricultural soils were at the range of 0.05–187 µg kg^−1^ DW [27,28,29]. Their persistence and accumulation in the soil are mainly attributed to their chemical binding and physical adsorption onto soil particles like clay minerals and humic acids [27,79,80]. However, MCs are poorly adsorbed to silty and sandy soils, and strongly retained in clayey soils, resulting in their fast and slow desorption, respectively; and thus, their availability to microbial degradation [81,82].

In this work, we noticed a decrease in MCs concentrations in non-sterile soil teemed with microorganisms in comparison to sterile soil devoid of microorganisms. These findings suggest that MCs might be partially removed via a microbially mediated degradation, which, thereby, resulted in less uptake of the toxin and less bioaccumulation in plants grown in non-sterile soil. On the same note, Cao et al. (2018) [59] carried out experimental trials regarding the fate of MCs, at the concentrations of 500 µg MC-LR kg^−1^, in sterile and non-sterile farmland soils under light and dark conditions. Data revealed that MCs have been fast-degraded, with a half-life of about 5 days, in soil inhabited by microorganisms with light and in the dark, concluding that microbially mediated degradation was the main pathway to alleviate MCs load in the soil rather than photodegradation. In our previous studies, we reported the advantages of the use of MC-rhizobia to alleviate the phytotoxic effects of MCs on faba bean (*V. faba*) and alfalfa (*M. sativa*) crops. The legume-rhizobia symbiosis enhanced plant physiological and biochemical response to MCs phytotoxicity, resulting in less severe growth impairments [42,43]. Rhizobial-mediated bioremediation seems to be an eco-effective and promising tool to mitigate MCs load in soils since rhizobia were found capable of degrading MCs as previously reported by Zhu et al. (2016) [83]. In this study, a novel isolate of *Rhizobium* sp. showed a high level of MC-degrading activity by hydrolyzing MC-LR from 8.3 mg L^−1^ to below the limits of detection within 10 h. Furthermore, Ramani et al. (2012) [84] have recorded that MCs were fast-degraded by about 74 % using a bacterial consortium consisting of two isolates *Rhizobium gallicum* and *Mycobacterium* sp. in batch culture. Moreover, in a previous study, we found out that the class of *betaproteobacteria* was stimulated and increased in abundance between bulk soil, rhizosphere, and root tissues of alfalfa (*M. sativa*) in response to MC exposure [85]. In line with this result, it has been previously indicated that a diverse group of bacterial genera, affiliated with *betaproteobacteria* class, were capable of degrading MCs, such as *Acidovorax*, *Methylotenera*, *Methylobacillus*, *Burkholderia*, *Ralstonia*, and *Paucibacter* [86,87,88,89,90].

It has to be pointed out that the MC-biodegradation process is highly influenced by soil physicochemical features. Soils with high content of organic matter stimulated the development of MC-degrading microbiota in a previous study, in which MCs (15 µg g^−1^) were removed within 7.1–17.8 days of half-life [27]. On another note, the biodegradation time of MCs, being mixed with agricultural soils, was reduced from 6 days to 4 days without any lag phase with the addition of humic acid [28]. Other authors reported that the MC-degradation process was stimulated with the addition of humic acid and NaNO_3_ to the soil. Although, the biodegradation rate was decreased when amending soil with NH_4_Cl, glycine, and glucose or using phytosanitary products such as glyphosate and chlorothalonil [59]. These findings suggest conducting further investigations on farm practices that may boost MC-degradation in soil-crop systems; and thus, prevent the toxin from impairing plant growth and threatening human and farm animal health.

Moreover, it has been proven that dissolved MCs (extracellular MCs) were rapidly degraded and removed by native microbiota in contrast to those preserved inside intact cyanobacterial cells (intracellular MCs). Intracellular MCs from cyanobloom biomass used as fertilizer were gradually released into the soil following cell breakdown and continuously taken up by the plants thereafter. This resulted in less biodegradation of the toxin, and, thereby, a high accumulation rate in plant tissues of the vegetable produce [40,91,92]. Furthermore, MC-producing cyanobacteria, affiliated with *Chroococcales*, *Oscillatoriales*, and *Nostocales*, have been detected in agricultural soils so far, which exacerbates the awkward situation related to MC accumulation in cropland soils [25]. However, it has been reported that bacterial strains isolated from soil and identified as *Bacillus* sp. were capable of exerting a lytic activity against *Microcystis aeruginosa* cells, besides degrading MCs-LR and -RR [93,94]. It has been also reported that MCs could be removed by adsorption and biodegradation processes by biofilms formed of bacteria and periphyton microalgae as shown in the study carried out by Wu et al. (2010) [95]. Therefore, we need to elaborate new bioremediation strategies based on the use of consortia consisting of soil lytic-cyanobacterium and MC-degrading microbial strains (bacteria and fungi) to remove MCs (intra- and extracellular) from cropland soils.

## 5. Conclusions

This study provided insights into the accumulation of MCs in plant tissues and their potential removal in bulk and rhizospheric soils. It is assumed throughout this work that rhizospheric microbiota reduced the bioavailability of MCs in soil matrix through a microbially mediated degradation. Therefore, plants grown in the absence of microorganisms evidenced severe growth impairments and accumulated high concentrations of MCs in edible tissues compared to those cultivated in presence of microorganisms. Native microbiota in the soil attenuated MC load within and, thus, decreased their uptake by the plants and thereafter diminished the sanitary risk over the consumption of the related contaminated tissues. In this light, screening of microorganisms capable of degrading MCs is recommended for further use in the soil-remediation process to reduce MC eco-risk and biohazard in cropland soils and related vegetable produce. It is also recommended to isolate these microbial strains from soils that are or were exposed to MCs for a relatively long term; since the probability to find highly effective MC-degrading strains within are higher than that of other soils.

## Figures and Tables

**Table 1:** Physical, chemical, and biological properties of the cropland soil used in the greenhouse trial. Data are presented as mean values ± standard deviation (*n* = 3).

Measured Parameters	Corresponding Values
**Physical Parameters**	
Particle-size distribution (%)	
Sand (50–2000 µm)	7.03
Silt (2–50 µm)	64.05
Clay (<2 µm)	28.92
Texture: Silty clay loam	
Clay minerals (%)	
Hartite	50
Wollastonite	20
Laumonite	11
Sillimanite	8
Clinoptilotite	7
Periclase	1
Moisture content (%)	12.65 ± 0.00
Water-holding capacity (%)	34 ± 1.00
**Chemical Parameters**	
pH (1:5 H_2_O)	8.17 ± 0.07
Electrical conductivity (dS m^−1^)	0.42 ± 0.06
Organic matter (g kg^−1^)	3.8 ± 1.00
Humic acids (mg g^−1^)	0.48 ± 0.02
Macronutrients (g kg^−1^)	
Nitrogen (N)	0.74 ± 0.15
Phosphorus (P)	0.42 ± 0.18
Potassium (K)	24.57 ± 0.42
**Biological Parameters**	
Total bacterial count	3.28 × 10^7^

**Table 2:** Growth indicators of faba bean (*V. faba* L., var. *Alfia 321*) and common wheat (*T. aestivum* L., var. *Achtar*) grown in sterile and non-sterile soils after 30-days exposure to MCs. Data are shown as mean ± standard deviation (*n* = 10); mean values are significantly different (*p* < 0.05) between the control (0 MCs µg L^−1^) and the treatment (100 MCs µg L^−1^) groups when indicated by (*).

Growth Indicators		MCs µg L^−1^	Plants/Treatments			
			Faba Bean		Common Wheat	
			Sterile Soil	Non-Sterile Soil	Sterile Soil	Non-Sterile Soil
Shoot	TLN (plant^−1^)	0	11 ± 1.00	12 ± 1.00	6 ± 0.00	6 ± 0.00
		100	11 ± 1.00	12 ± 1.00	6 ± 0.00	6 ± 0.00
		% Change	0.00	0.00	0.00	0.00
	SL (cm plant^−1^)	0	38.66 ± 1.07	32.28 ± 1.31	23.02 ± 0.48	24.47 ± 0.26
		100	32.12 ± 1.76 *	32.03 ± 1.53	20.03 ± 0.69 *	24.4 ± 0.21
		% Change	16.90	0.80	13.00	0.30
	SDW (g plant^−1^)	0	0.8 ± 0.04	0.62 ± 0.04	0.24 ± 0.02	0.21 ± 0.00
		100	0.62 ± 0.03 *	0.67 ± 0.04	0.16 ± 0.01 *	0.214 ± 0.01
		% Change	22.39	−8.36	33.05	−1.90
Root	LRN and FRN (plant^−1^)	0	36.3 ± 2.50	34.7 ± 1.20	7 ± 0.00	7 ± 0.00
		100	23.7 ± 3.00 *	34.7 ± 3.20	6 ± 0.00	8 ± 0.00
		% Change	34.71	0.58	14.29	−14.29
	RL (cm plant^−1^)	0	21.68 ± 1.81	18.15 ± 0.82	23.72 ± 0.89	23.8 ± 1.73
		100	16.38 ± 0.53 *	18.2 ± 1.40	20.03 ± 0.62 *	22.85 ± 1.42
		% Change	24.45	−0.28	15.60	4.00
	RDW (g plant^−1^)	0	0.28 ± 0.02	0.24 ± 0.05	0.025 ± 0.00	0.031 ± 0.00
		100	0.19 ± 0.04 *	0.23 ± 0.04	0.018 ± 0.00	0.029 ± 0.00
		% Change	33.10	3.36	26.98	6.54

TLN: total leaf number; SL: stem length; SDW: shoot dry weight; LRN: lateral root number (faba bean); FRN: fasciculated root number (common wheat); RL: root length; RDW: root dry weight.

**Table 3:** MC content in bulk soil and soil-plant system, and their bioaccumulation factor (BAF) in shoot and root tissues of faba bean (*V. faba* L., var. *Alfia 321*) and common wheat (*T. aestivum* L., var. *Achtar*) plants. Data are shown as mean ± standard deviation (*n* = 3). The bioconcentration level (BL) was evaluated based on BAF values.

MC Content (µg kg^−1^ DW)	Planted Soil				Unplanted Soil (Bulk Soil)	
	Faba Bean		Common Wheat			
	Sterile Soil	Non-Sterile Soil	Sterile Soil	Non-Sterile Soil	Sterile Soil	Non-Sterile Soil
Shoot	7.65 ± 0.39	6.99 ± 0.02	88.12 ± 5.61	60 ± 7.73	-	-
BAF (shoot)	0.08 ± 0.00	0.07 ± 0.00	0.88 ± 0.06	0.6 ± 0.08	-	-
BL	Low	Low	Low	Low	-	-
Root	49.13 ± 3.88	20.38 ± 0.88	12.77 ± 8.48	5.47 ± 0.49	-	-
BAF (root)	0.49 ± 0.04	0.2 ± 0.01	0.13 ± 0.08	0.05 ± 0.00	-	-
BL	Low	Low	Low	Low	-	-
Rhizospheric/bulk soil	0.187 ± 0.04	0.11 ± 0.00	0.117 ± 0.07	0.08 ± 0.00	0.161 ± 0.09	0.114 ± 0.02

DW: dry weight.

**Table 4:** Estimated daily intake (EDI; µg eq MCs kg^−1^ of bw d^−1^) and health risk quotient (RQ) values for humans and livestock (cattle). Data are shown as mean ± standard deviation (*n* = 3). Risk level was assessed based on health risk quotient (RQ) values. Bolded values are those higher than the tolerable daily intake (TDI) set at 0.04 and 0.45 µg kg^−1^ of body weight day^−1^ for human and cattle, respectively.

Health Risk Parameters	Faba Bean				Common Wheat			
	Human		Cattle		Human		Cattle	
	Sterile Soil	Non-Sterile Soil	Sterile Soil	Non-Sterile Soil	Sterile Soil	Non-Sterile Soil	Sterile Soil	Non-Sterile Soil
EDI	4.3 × 10^−3^ ± 0.00	4 × 10^−3^ ± 0.00	**0.31 ± 0.02**	**0.28 ± 0.00**	**0.73 ± 0.05**	**0.5 ± 0.06**	**3.52 ± 0.22**	**2.4 ± 0.31**
RQ	0.11 ± 0.00	0.1 ± 0.00	0.68 ± 0.03	0.62 ± 0.00	18.36 ± 1.10	12.5 ± 1.60	7.83 ± 0.50	5.33 ± 0.70
Risk level	Moderate	Moderate	Moderate	Moderate	High	High	High	High

## Data Availability

Data regarding average daily intake of vegetables (used to calculate the estimated daily intake of microcystins) can be obtained from the Food and Agriculture Organization of the United Nations’ website at: http://www.fao.org/faostat/en/?#data/FBS, accessed on 16 August 2021.
